# The Development of Formulaic Knowledge in Super-Advanced Chinese Language Learners: Evidence From Processing Accuracy, Speed, and Strategies

**DOI:** 10.3389/fpsyg.2022.796784

**Published:** 2022-03-03

**Authors:** Hang Zheng, Bo Hu, Jie Xu

**Affiliations:** ^1^Department of Chinese (Zhuhai), Sun Yat-sen University, Zhuhai, China; ^2^Faculty of Humanities and Social Sciences, City University of Macau, Macao, Macao SAR, China; ^3^Faculty of Arts and Humanities, University of Macau, Macao, Macao SAR, China

**Keywords:** language proficiency, formulaic sequence, processing strategy, think-aloud, CSL (Chinese as a second language)

## Abstract

The study examined the development of Chinese as a second language learners’ formulaic knowledge through comparing the processing of Chinese idioms versus non-idiomatic formulaic sequences (FSs) by advanced-level learners (ALs), super-advanced learners (SLs), and native speakers (NSs). Using two phrase acceptability judgment tasks with and without think-aloud protocols, we collected data on participants’ processing accuracy, processing speed, and processing strategies of reading the two types of FSs. Four processing patterns emerged from the analyses of the datasets. First, learners’ processing accuracy and speed increased along with their proficiency. Second, learners’ idiom processing ability was generally lower than that of non-idiom processing ability, but they demonstrated an improving trend as their proficiency level increased. Third, learners’ use of processing strategies did not change much as proficiency rose and demonstrated a categorical difference from NSs. Fourth, all three groups exhibited poorer productive idiom knowledge than productive non-idiom knowledge. The overall findings denote that second language learners’ formulaic knowledge can evolve beyond the lexical plateau as learners move from the advanced to a higher proficiency level, but the productive idiom knowledge can be a long-term problem. The findings provide implications for measuring and teaching Chinese formulaic knowledge at the higher-than-advanced stage.

## Introduction

In second language acquisition (SLA), the two primary goals are to identify the nature of learners’ linguistic knowledge and to provide accounts for how this knowledge transforms over time (Ellis, 2005; Bowles, 2011). Specifically for research on Chinese as a second or foreign language (CSL/CFL), Gong et al. (2020b) pointed out that it is important to measure students’ learning outcomes and to gauge the underlying mechanisms that impact the development of students’ language proficiency.

In the last two decades, mounting evidence has suggested that overall second language (L2) proficiency can be improved by acquiring formulaic sequences (FSs), which are multiword expressions that frequently recur as a whole in language use (Biber and Conrad, 1999; Wray, 2000; Cortes, 2015; Wood, 2019). This claim has been supported by corpus linguistic, psycholinguistic, and functional linguistic research (Schmitt, 2010; Myles and Cordier, 2017). Corpus linguistic research indicates that a small class of FSs covers a fairly large portion of spoken and written texts; this ubiquity underscores the importance of FSs in the use of language (e.g., Oppenheim, 2000; Bestgen, 2017). The psycholinguistic research shows that native speakers (NSs) process FSs more efficiently than they process rule-generated phrases (e.g., Jiang and Nekrasova, 2007; Siyanova-Chanturia et al., 2011), suggesting that FSs are likely to be prestored and retrieved as whole units directly from long-term memory. Functional linguistic research indicates that FSs are semantically integral, which allows for the relatively complex meaning and function to be compacted into somewhat simple sequences. Hence, learning FSs can make communication more sophisticated and efficient (Schmitt and Carter, 2004; Tang and Taguchi, 2021). These findings led to the assumption that learning FSs can facilitate language comprehension and production (Boers et al., 2006; Lindstromberg and Boers, 2008; Taguchi et al., 2013; Yan, 2020; Saito and Liu, 2021). As such, mastering FSs can lead to the development of overall language proficiency (Weinert, 1995; Wray, 2000).

Despite the general acknowledgment of the importance of learning formulaic knowledge, little is known about how formulaic knowledge develops as learners’ proficiency increases. Some corpus-based studies (e.g., Qi and Ding, 2011; Staples et al., 2013) focused on portraying the change of FS use by learners at different proficiency levels, but did not tap into learners’ cognitive processes that may regulate such change. This study set out to fill the gap by comparing how advanced learners (ALs), super-advanced learners (SLs), and NSs process two types of Chinese FSs. Based on the patterns of processing accuracy, processing speed, and processing strategies, we attempted to investigate the development of L2 learners’ formulaic knowledge as well as the underlying mechanisms that may impact the development.

## Literature Review

In the FS literature, there is a severe lack of studies gauging the development of L2 formulaic knowledge. This scarcity of research may have to do with certain characteristics of FSs. We review these characteristics and the related empirical findings in the following sections.

### Formulaic Sequence Types

Formulaic sequences lack homogeneity. Different types of FSs vary greatly in their linguistic dimensions, such as idiomaticity, form fixedness, function, and linguistic register (Wray and Perkins, 2000). For example, idioms (e.g., “kick the bucket”), lexical collocations (e.g., “kick the ball”), and lexical bundles (e.g., “one of the most”) are all formulaic expressions. However, the three kinds of FSs are quite different in the degree of semantic transparency. Idioms have low degree of semantic transparency; their conventional meanings are often different from their literal interpretations. Thus, idioms often cause problems to language comprehension because their meanings might not be derived from analyzing the constituent words and their grammatical relations (Cacciari and Tabossi, 1988). In contrast, lexical collocations and lexical bundles are highly transparent; their meanings are unlikely to “cause trouble for L2 learners from a comprehension perspective” (Wolter and Yamashita, 2018, p. 396). In this sense, lexical collocations and lexical bundles are similar to non-formulaic novel phrases; one thing that sets them apart is that lexical collocations and lexical bundles are recurrent in language use, enjoying significantly higher frequency than novel phrases do (Jiang and Nekrasova, 2007).

In NSs’ FS processing, although researchers generally agree that FSs have a processing advantage over non-formulaic novel phrases, Carrol and Conklin (2020) found that the factors contributing to the processing advantage of different types of FSs were fundamentally different, suggesting that the processing of FSs is type-sensitive.

In L2 learners’ FS processing, studies focusing on different kinds of FSs have often reported different processing patterns. For example, by examining idiomatic FSs, Conklin and Schmitt (2008) employed a self-paced reading paradigm to compare the processing of idioms’ literal interpretation (“take the bull by the horns” interpreted as “wrestle with an animal”) to their figurative interpretation (“take the bull by the horns” interpreted as “to attack a problem”). They found a processing advantage of idioms’ figurative reading among both NSs and learners. However, when Siyanova-Chanturia et al. (2011) harnessed eye tracking to test NSs’ and learners’ idiom processing in literal versus figurative contexts, they did not detect any idiom processing advantage among learners due to their prolonged processing time spent on the idioms’ figurative reading before reaching the idiom key, where an idiom can be recognized as an idiom. When targeting non-idiomatic FSs, Jiang and Nekrasova’s (2007) grammaticality judgment tasks showed that both NSs and learners processed non-idiomatic FSs (“to tell the truth”) significantly faster than their matched novel sequences (“to tell the price”) with fewer errors. However, in series studies exploring another type of non-idiomatic FS— lexical collocations using eye-tracking paradigms (Vilkaitė, 2016; Vilkaitė and Schmitt, 2019)—the authors found that NSs and learners both processed adjacent collocations (“provided information”) significantly faster than their free combination controls, while only NSs processed non-adjacent collocations (“provide some of the information”) faster than free combinations. However, while scrutinizing lexical collocations in grammaticality judgment tasks, Gyllstad and Wolter (2016) reported a contrary processing pattern where both NSs and learners processed lexical collocations significantly more slowly than they processed free word combinations.

Due to conflicting findings, researchers have recently begun to call attention to the necessity of empirically comparing different types of FSs in SLA-oriented studies to discern the sources of learners’ processing difficulties, and to better integrate findings about FS processing into FS acquisition (Myles and Cordier, 2017; Carrol and Conklin, 2020). The most successful attempts can be found in a series of studies that aimed to identify L1 to L2 transfer occurring in the idiom domain (see Carrol et al., 2016 for a review) and the non-idiomatic collocation domain (see Wolter and Yamashita, 2018 for a review). By comparing the processing of L1-L2 congruent and incongruent FSs, these studies indicated that incongruency (an expression existing in the L1 but not in the L2, or vice versa) is a major source of FS processing difficulty, which to different degrees interacts with how frequently learners may encounter the FSs in the input (objective frequency) and how familiar they are with the FSs (subjective frequency); in turn, this affects their processing accuracy and processing speed. The pedagogical implication of these findings is that L2 learners may acquire different types of FSs through diverse sources and at different stages of learning (Wood, 2019). Hence, it is unlikely to draw a reliable conclusion about how L2 learners’ formulaic knowledge develops when FS is used as an “umbrella term” (Myles and Cordier, 2017).

### Chinese Formulaic Sequences

Chinese FSs share the universal characteristics that have been observed in FSs of other languages. They also lack homogeneity, and studies on the processing of different types of Chinese FSs by CSL learners have also reported conflicting results. For example, Zheng et al. (2016) used a grammaticality judgment task and a self-paced reading task to compare Chinese NSs’ and learners’ processing of a mixed class of FSs presented in isolation versus embedded in context. When FSs were presented in isolation, both NSs and L2 learners demonstrated significant FS processing advantages over the control novel phrases. However, when FSs were embedded in sentences, the processing advantage disappeared from the non-native speaker group. The authors concluded that the nature of L2 learners’ and NSs’ FS knowledge may be fundamentally different due to the different FS learning contexts. In contrast, using eye-tracking paradigms, Yi et al. (2017) explored the effects of whole-phrase frequency and constituent words’ co-occurrence probability in the online processing of Chinese adverbial FSs embedded in sentences. They found that both NSs and L2 learners were sensitive to phrasal frequency and co-occurrence probability. Based on this processing pattern, the authors concluded that NSs and adult L2 learners may share the same statistical learning mechanism despite the different FS learning contexts.

Among all types of FSs, Chinese idioms, such as 成语 Cheng-yu *set-language* (e.g., “杯弓蛇影” *cup-bow-snake-shadow* “to mistake the shadow of a bow projected in one’s cup as the shadow of a snake, indicating a false alarm or self-created suspicion”) and 惯用语 Guan-yong-yu *habitual-use-language* (e.g., “翘辫子” *rise-braid-SUFFIX* “to be dead”), may be the thorniest type for L2 learners. Chinese idioms are often “composed in Classical Chinese and thus typically have a different grammatical structure from that of Modern Chinese” (Jiao et al., 2013, p. vi). Moreover, Chinese idioms often have highly compact meanings because of the limited length of three or four characters. Due to their classical origin and compact meaning, the acquisition of Chinese idioms can be particular difficult, and the proper use of Chinese idioms are often considered an “important indicator of one’s overall language proficiency” (Li and Tat, 2014, p. 338). For CSL/CFL teachers, idioms are categorized as a “luxury” component for Chinese learners (Guo, 2017; Yang, 2020). The empirical research has also found that L2 learners’ idiom knowledge is often incomplete. Zheng et al. (2022) found that some idioms can only be correctly recognized but cannot be correctly understood or used. Duff and Li (2004) found that the speech quality of advanced Chinese learners who already have very good speaking and listening skills is highly constrained due to the improper use idioms. In other words, Chinese idioms represent very advanced knowledge that is particularly difficult to grasp, but they cannot be circumvented if near-native proficiency is to be achieved. In fact, idioms in other languages are also considered the steppingstone to achieve higher-than-advanced proficiency (Kim, 2016). Unfortunately, this proficiency can be attained by only a very small portion of learners (Webb, 2018).

### Proficiency Levels and Formulaic Sequence Processing by L2 Learners

#### Proficiency Levels

In SLA contexts, proficiency levels can be operationalized by more practical or theoretical approaches. In educational settings, more practical approaches are often adopted. College-level foreign language programs tend to use the seat time to determine the proficiency status. By this criterion, the advanced proficiency often requires three to four semesters in the language program (Byrnes, 2012, p. 510), and the higher-than-advanced proficiency would need additional time. The discipline programs often specify proficiency levels through standardized test scores, such as the Oral Proficiency Interview (OPI), derived from the American Council on the Teaching of Foreign Languages Proficiency Guidelines-Speaking (American Council on the Teaching of Foreign Languages [ACTFL], 2012), and the Test of English as a Second Language (TOEFL). Based on performance descriptors for the TOEFL iBT, advanced learners would score 24–30 in the reading section, 22–30 in the listening section, 25–30 in the speaking section, and 24–30 in the writing section (Educational Testing Service [ETS], 2021). The ACTFL guidelines distinguish the advanced and superior levels of oral proficiency. The major difference between advanced-high level and superior level is that advanced-high speakers “cannot sustain performance at that level across a variety of topics” (American Council on the Teaching of Foreign Languages [ACTFL], 2012, p. 5). As an instrument for evaluating the functional language ability, the ACTFL proficiency guidelines share the same theoretical framework with the systemic functional linguistic approach, basing L2 proficiency on learners’ abilities to use the target language in various genres (Ryshina-Pankova, 2018). From the psycholinguistic perspective, L2 proficiency is determined by the extent to which the late L2 learners (beyond the critical period) can process the target language in a native-like manner (Roberts, 2018). Because NSs command a wide range of formulaic language and advanced learners do not (Pawley and Syder, 1983), formulaic language was often used in psycholinguistic studies to address the proficiency effect (Bui et al., 2018).

#### The Proficiency Effect on L2 Learners’ Formulaic Sequence Processing

Due to the difficulty in recruiting highly proficient L2 learners who have a sufficiently large FS repertoire to gauge the formulaic development, only a few studies on L2 FS processing have addressed the proficiency effect. Nekrasova (2009) compared intermediate and advanced English learners’ and English NSs’ knowledge status of two FS types: discourse-organizing bundles (e.g., “what do you think”) and referential bundles (e.g., “one of the most”). The purpose was to determine whether these FSs are perceived as holistic units. To achieve that goal, Nekrasova conducted a gap-filling task to measure speakers’ precise knowledge of the constituent words in FSs, and a dictation task to gauge the participants’ knowledge of the holistic forms of the FSs. On the gap-filling task, intermediate learners scored significantly lower than the other two groups, while there was no significant difference between advanced learners and NSs. Based on this finding, the author concluded that learners who are capable of accurately producing FSs develop their skill as their proficiency increases. However, on the dictation task, advanced learners outperformed both intermediate learners and NSs in the amount and accuracy of the FSs that they recalled. The author speculated that lower-proficiency learners may have insufficient FS knowledge, while NSs may have an FS repertoire that is too large, which probably led to both groups’ poor performance in recalling the exact forms of the dictated FSs. In addition, the comparisons of the two FS types revealed that all three groups acquired discourse organizers better than referential bundles on both tasks; this implied that the speakers’ knowledge of FSs was affected by linguistic register and discourse function. Given the complicated outcomes, the author cautioned that more than one measurement should be used to determine the psychological validity of a particular FS type. In a more recent study, Wolter and Yamashita (2018) compared how intermediate English learners, advanced English learners, and English NSs processed L1 (Japanese)-L2 (English) congruent and L1-L2 incongruent collocations, and whether their processing speed was impacted by constituent word frequency and collocational frequency. In a phrase acceptability judgment task, the learners read four types of adjective-noun collocations under congruency conditions: (1) congruent; (2) English-only incongruent; (3) Japanese-only incongruent; and (4) non-collocational free phrases. Wolter and Yamashita analyzed the reading times of the items that were correctly responded to. In contrast to NSs, they found that L2 learners processed congruent collocations significantly faster than they processed English-only collocations. The authors attributed the congruency effect to the age of acquisition. For the proficiency effect, although all three groups exhibited sensitivity to both word-level and whole-form-level frequency, as learners’ proficiency increased, they experienced a shift from greater sensitivity to word frequency to greater sensitivity to collocational frequency, representing a development toward nativeness. Based on this developmental pattern, the authors rebutted previous claims that there is a fundamental difference between NSs and L2 learners’ processing of FSs, arguing that FS processing behavior evolves as speakers’ language proficiency increases. From the methodological perspective, both studies showed that the development pattern could not be observed without the fine-grained manipulation of FS materials.

Also, concerning the research methods, although both studies addressed the proficiency effect in L2 learners’ FS processing, Nekrasova (2009) used controlled production tasks, while Wolter and Yamashita (2018) employed online perception tasks. Controlled production tasks tap into productive knowledge, and online perception tasks tap into receptive knowledge. However, previous research has pointed out that the development of learners’ receptive knowledge and that of their productive knowledge are often unbalanced (Laufer, 1998; Daller et al., 2013). Therefore, to gain a more comprehensive view of how L2 learners’ formulaic knowledge develops, both types of knowledge need to be investigated.

The current study went a step further, triangulating online perception data and unstructured production data by using a speedy phrase acceptability judgment task and a phrase acceptability judgment task with think-aloud protocols. The speedy task collects participants’ yes-or-no judgments and response time, through which we can generalize FS processing patterns (Jiang, 2013). The think-aloud task gathers participants’ thought processes, through which we can infer what processing strategies the participants use (Leow et al., 2014; Kim and Bowles, 2019). The overall processing patterns and detailed processing strategies can complement each other and provide a fuller picture of how the status of FS knowledge may evolve as proficiency level increases. The three research questions (RQs) that guided the study are as follows:

(1) Do ALs, SLs, and NSs demonstrate different patterns in FS processing accuracy?
(2) Do ALs, SLs, and NSs demonstrate different patterns in FS processing speed?
(3) Do ALs, SLs, and NSs demonstrate different patterns in their FS processing strategies?

## Materials and Methods

### Participants

#### Recruitment

The participants included 13 ALs, 13 SLs, and 13 NSs. Although a larger sample size is always desirable for lexical research to iron out individual variances, Schmitt (2010) pointed out that “for psycholinguistic tasks with very precise measurement, this may require only 10 or 20 subjects” (p. 150). In the initial recruitment stage, 30 non-heritage CSL degree pursuers who had passed the highest level (Level 6) of the standardized Chinese language proficiency test (**HSK:** ***H**angyu* ***S**huiping* ***K**aoshi*) volunteered to participate. Before the main test, the participants took an online character quiz designed and distributed by the APP Wenjuanxing questionnaire. On the quiz, the participants were asked to associate each character with its correct meaning on a multiple-choice task. The characters included (but were not limited to) all the characters that would appear in the testing materials. All characters were within the required vocabulary range of the HSK 6 guidelines, so presumably the participants would already know all the characters. Knowing all target characters is important because it can confirm that any observed processing problems are NOT due to the presence of unknown words in the FSs. Finally, we included 26 learners (mean age = 26) who achieved a score of 100% on the quiz on the main test. Another 13 NS participants were undergraduate and graduate students recruited from two universities in Beijing (mean age = 27). None of them were Chinese language or literature majors.

#### Proficiency Levels of the L2 Participants

The 26 L2 participants came from four Chinese universities and were enrolled in three types of programs: (1) language degree programs designed for foreign students to learn Chinese for general and specific purposes; (2) discipline programs (including psychology, biology, economics, Chinese language and literature, and foreign language and literature) that mostly enroll Chinese undergraduate students and some very advanced foreign students who are able to use Chinese in academic settings; and (3) preparatory programs designed to prepare advanced foreigners to enter into discipline-focused programs. The background information about the L2 participants is presented in Table 1.

**TABLE 1:** Background information of L2 participants.

Ranking	Subject	Age	HSK score	Years of learning	Nationality	Program
Top 13 scorers	1	23	273	10	Korea	Discipline
	2	24	270	10	Korea	Discipline
	3	29	264	4	Korea	Discipline
	4	22	260	10	Korea	Preparatory
	5	19	250	7	Korea	Discipline
	6	26	248	3	Vietnam	Discipline
	7	22	247	9	Korea	Discipline
	8	24	246	9	Korea	Preparatory
	9	24	240	7	Korea	Preparatory
	10	30	232	8	Thai	Discipline
	11	21	232	10	Mongolia	Language
	12	31	230	5	Korea	Preparatory
	13	33	229	7	Thai	Discipline
Bottom 13 scorers	14	28	220	3	Korea	Language
	15	23	220	5	Kazakhstan	Preparatory
	16	27	220	6	Egypt	Language
	17	31	219	7	Korea	Language
	18	32	218	5	Russia	Preparatory
	19	23	212	2	Korea	Language
	20	27	210	4	Japan	Language
	21	30	207	6	Kazakhstan	Language
	22	27	204	6	Egypt	Language
	23	24	194	6	Kazakhstan	Preparatory
	24	23	188	6	Japan	Language
	25	25	187	4	Japan	Language
	26	29	186	2	Korea	Language

We further divided the 26 learners into two proficiency groups. Because the HSK test does not have official guidelines to differentiate advanced and higher-than-advanced levels of proficiency, we used learners’ HSK scores as the primary criterion to distinguish their proficiency levels. We grouped the 13 highest scorers together (mean score = 247.77) and the 13 lowest scorers together (mean score = 206.54). Independent *t*-tests confirmed that the two groups’ HSK scores were significantly different (*df* = 24, *t* = −7.31, *p* < 0.000). Other than HSK scores, we also considered years of learning the language and current enrollment programs. Shohamy (2006) proposed that the “advancedness” of language proficiency can be assessed by the extent to which language serves as a means for content knowledge learning, or if language itself is the goal of instruction. Based on this criterion, the top 13 scorers who were primarily learners in discipline-focused programs (*n* of learners in a discipline-focused program = 8, *n* of preparatory program learners = 4, *n* of language program learners = 1) would be considered more advanced than the bottom 13 scorers, who were mostly learners in language programs (*n* of learners in a language program = 10, *n* of learners in a preparatory program = 3). Moreover, Byrnes et al. (2010) argued that an undisputed characteristic of advanced language capacity is the long-term cumulative nature. Given the number of years it takes to learn Chinese, the top 13 scores (mean years of learning = 7.62) were more advanced than the bottom 13 scores (mean years of learning = 4.77). Hence, we identified the top 13 scorers as super-advanced learners and the bottom 13 scorers as advanced learners.

### Test Materials

The two types of FSs used in this study were Chinese idioms and non-idiomatic FSs. Because we differentiated FSs by the domain of idiomaticity, we treated subtypes of non-idiomatic FSs (i.e., lexical collocation and lexical bundles) as one category. For both idioms and non-idioms, we also manipulated the sequence length by using three-character (3-C) sequences and four-character (4-C) sequences with matched whole-form frequency. The rationale is that because 4-C items are one character longer than 3-C items, if 3-C items are processed significantly faster than 4-C items of the same FS type, then it is very likely that the FSs will be visually recognized in a word-by-word fashion. If the length does not play a significant role in processing speed, then it is more likely that the FSs will *not* be read in a verbatim fashion.

We chose the target idioms based on the following steps. First, we manually extracted all 3-C and 4-C idioms listed in *The Contemporary Chinese Dictionary* (6th edition). We tried to be as exhaustive as possible. This procedure yielded a total of 2,098 4-C idioms and 3,783 3-C idioms. The 4-C idioms are all 成语 Cheng-yu; the 3-C idioms include both 惯用语 Guan-yong-yu and semantically non-transparent, verb-complement structures (e.g., 看上去 look-up go “*it seems like*”). Second, we ran the two types of idioms in the Google 1-gram database. Given the raw token frequency, we further extracted the top-ranked 300 items in each type. Then, by consulting with two experienced Chinese language teachers, we selected 38 3-C and 38 4-C idioms that are more likely to be familiar to advanced learners and have comparable whole-form frequencies for the idiom pool. Finally, using a 5-point scale, 33 Chinese language teachers rated these idioms based on the likelihood that an advanced Chinese learner would know them. Finally, we included 24 3-C idioms and 24 4-C idioms that received an average score of 4 or higher (see Supplementary Materials Table A for the ratings). The Cronbach’s alpha coefficient (0.748) suggests that the raters’ internal consistency was acceptable.

Because we aimed to compare the processing of idioms and non-idiomatic FSs, we matched each idiom with non-idiomatic FSs through the following steps. We first changed one constituent word of the target idiom and waited to see if the new expression would be grammatical, and if the whole-form frequency and total stroke number were matched with the target idiom (e.g., 大吃一惊 big-eat-one-surprise “be astounded at” vs. 大吃一顿 big-eat-one-meal “eat a big meal”). If changing one character failed, we continued to change two characters (e.g., 心中有数 heart-middle-have-number “have a clear ideal about” vs. 心里有事 heart-in-have-thing “have something in mind”). If changing two characters also failed, we used the fuzzy search function provided in the BCC corpus and looked for a potentially non-idiomatic expression that shared at least one content character with the target idiom (e.g., 前所未有 before-place-not-have “unprecedented” vs. 不可能有 not-can-able-have “cannot have”). After several rounds of matching, we found that some of the chosen non-idioms were absent from the existing Chinese frequency corpora. To solve this problem, we adopted Libben and Titone’s (2008) method using the most popular Chinese website search engine^1^ as the database, and we employed the log-transformed page count to represent the whole-form frequency. The selected non-idioms fulfilled the following criteria: (1) they are grammatical phrases commonly used in modern Chinese; (2) they have equal numbers of characters and similar constructions to the matched idioms; (3) they share at least one identical content character with the matched idioms; and (4) they have comparable total stroke numbers and whole-form frequency to the matched idioms. Independent samples *t*-tests (*a* = 0.0125) showed no significant differences in the 3-C idiom and 3-C non-idiom frequencies (*t* = −0.57, *df* = 46, *p* = 0.57) or stroke numbers (*t* = 0.36, *df* = 46, *p* = 0.72). The 4-C idiom and 4-C non-idiom frequencies were also not significantly different (*t* = 0.09, *df* = 46, *p* = 0.93), nor were their stroke numbers (*t* = 0.08, *df* = 46, *p* = 0.94). The Independent samples *t*-tests also confirmed that the 24 3-C idioms and 24 4-C idioms were matched in whole-form frequency (*t* = −1.49, *df* = 46, *p* = 0.14), as were the 24 3-C non-idioms and 24 4-C non-idioms (*t* = 1.69, *df* = 46, *p* = 0.72).

In sum, the test stimuli (in Supplementary Materials Table A) consisted of four categories of FSs: (1) 3-C idioms (*n* = 24) and (2) matched 3-C non-idioms (*n* = 24) and (3) 4-C idioms (*n* = 24) and (4) matched 4-C non-idioms (*n* = 24). Another 96 ungrammatical phrases were made up as fillers. We divided the test stimuli into two counterbalanced blocks so that the participants would not read an idiom and its matched non-idiom in the same block.

### Instruments

#### The Speedy Phrase Acceptability Judgment Task

We programmed the speedy acceptability judgment task using Paradigm software to collect judgment accuracy data and response time data. Acceptability judgment task paradigms have been widely used in the SLA field to measure learners’ L2 competence (e.g., Ellis, 1991; Han and Ellis, 1998; Loewen and Erlam, 2006). Because the time pressure imposed by speedy/timed tasks reduces the chance of accessing metalinguistic knowledge, speedy acceptability judgment tasks are often considered a measure of implicit knowledge (Ellis, 2005; Bowles, 2011) and the amount of cognitive effort spent (Jiang, 2013). For some linguistic phenomena (i.e., FS) that are not easily elicited in free production tasks, the acceptability judgment task is also more practical because it can test a large number of target forms and can therefore gather more representative data. For this task, the participants were informed that they would see some Chinese phrases and that they needed to indicate whether a phrase is likely to be read or heard in Chinese. A phrase was presented on a computer screen after an 800-millisecond (ms) presentation of a fixation cross; the participants were asked to press the “A” key if the phrase seemed acceptable in Chinese or the “L” key if the phrase seemed unacceptable, as quickly as they could. The phrase remained on the screen until a response key was pressed. The participants then took a 5-min break and returned to complete the second block.

#### The Think-Aloud Phrase Acceptability Judgment Task

We conducted the think-aloud phrase acceptability judgment task 1 week after each participant finished the speedy acceptability judgment task. This task allowed us to examine participants’ thought processes when they judged the phrases. The purpose was to infer what processing strategies were used by different groups of participants and for different types of FSs. As a versatile data collection tool, the think-aloud method has been broadly employed in L2 studies to gather data about learners’ thought processes to measure processing strategies (Cooper, 1999), depth of processing (Kim and Bowles, 2019), or linguistic awareness (Leow and Morgan-Short, 2004). In mixed-methods research, think-aloud protocols are harnessed to complement other concurrent data collection procedures to obtain nuanced information that quantitative methods might not be able to capture (Leow et al., 2014).

In previous SLA research, think-aloud protocols can be classified into two types—metalinguistic and non-metalinguistic—each tapping into different kinds of knowledge and having different techniques. The metalinguistic think-aloud protocols—which are carried out by asking participants to provide justifications for their performance—tap into metalinguistic knowledge. The non-metalinguistic think-aloud protocols—which are conducted by simply asking the participants to verbalize whatever is on their minds while performing a task—tap into general cognitive processes (see Bowles, 2010, Chapter 2 for a thorough review). Since it is impossible for participants to vocalize all of their thoughts, neither of the two approaches can verify whether the non-metalinguistic think-alouds represent a more accurate reflection of learners’ thought processes, because the non-metalinguistic instruction does not ask participants to explain their performance, and thus does not intentionally induce participants to access any particular type of knowledge (Ellis and Loewen, 2007). The disadvantage of non-metalinguistic think-alouds is that think-aloud data provide enough details, but not all of them are directly related to the research questions.

For this study, we generally used a non-metalinguistic approach, asking participants to report whatever came to their mind when making a yes-or-no judgment. However, instead of emphasizing “don’t explain your thoughts” (Sanz et al., 2009, p. 53), the participants were asked to answer, “How do you know that the phrase is acceptable or not?” without requiring a specific type of information. We conducted two rounds of pre-tests and found that this instruction did not especially induce the participants to provide metalinguistic information. For example, on the pre-test, some participants simply reported that “我就感觉是对的 *I just feel it’s correct*” or “我听过这个 *I’ve heard of this.*” On the test, we used the same set of test stimuli and a different set of filler stimuli. The test stimuli were presented one by one on a computer screen controlled by a research assistant. Because L2 learners were also asked to verbalize in Chinese, they were informed that they need not worry about language errors, because the purpose of the task was to understand their thought process, rather than testing verbal proficiency (Kim and Bowles, 2019). Adapted from Sanz et al. (2009, p. 53), the instructions were as follows:

“请判断这个短语在汉语里能不能说，请说出您是怎么知道的。就把您思考时在脑子里对自己说的话大声说出来，就好像您独自坐在一个房间里跟自己说话一样。不用在意说的句子语法对不对，完整不完整，因为我们只想了解您在做判断时的想法。 (*Please judge whether or not this phase is acceptable in Chinese, and state how you know that. Just say out aloud everything that you would say to yourself silently while you think. Just act as if you were sitting alone in the room speaking to yourself. Don’t worry about whether your sentence is grammatical or complete because we only want to know about your thought process while making the judgment.)*”

We used five grammatical FSs and five ungrammatical phrases in the practice session to ensure that the participants were able to think aloud appropriately. On the main test, to ensure that participants’ thinking aloud was constant throughout the experiment, they were reminded to keep talking when they fell silent. We audio-recorded the entire think-aloud session using Audacity and manually transcribed the data.

### Coding

We coded the participants’ think-aloud reports for processing strategies. In this study, “processing strategy” refers to what type of information a participant relied on to make a judgment. A participant could use his/her intuition and say, “*I just feel it is correct*,” or think of an example sentence, such as “看热闹,对的,两个人打架我去看热闹. (‘*Kan-re-nao,’ correct. Two men are fighting and I go to watch the fun*)”. When developing the coding book, we consulted one grammaticality judgment study and one idiom processing study. In Rebuschat and Williams’ (2012) study, participants were asked to report what source of knowledge they used to make grammaticality judgments, and the verbal reports were coded into five categories: guess, intuition, pre-existing knowledge, rules, and memory. In Cooper’s (1999) study, L2 learners were asked to verbalize what they were thinking while reading a passage that included idioms. Learners’ idiom processing strategies were coded into eight categories: repeating or paraphrasing; analyzing the context; requesting information; guessing the meaning; using the literal meaning of the idiom; using background knowledge; referring to one’s L1; and other strategies. Referring to the above two coding schemes and our preliminary observations of the think-aloud data, we created five categories for processing strategies: (1) using intuition (abbr. intuition); (2) thinking of examples (abbr. example); (3) making an interpretation (abbr. interpretation); (4) associating pre-existing knowledge (abbr. association); and (5) performing metalinguistic analysis (abbr. metalinguistics). Table 2 presents the think-aloud evidence for each processing strategy.

**TABLE 2:** Processing strategies, specific presentations, and TA evidence.

Strategy	TA evidence
**Intuition**	
*Feeling*	我听起来就不对.
	It doesn’t sound right to me.
*Confession*	我见过这个，可是意思忘了.
	I have seen this one, but forgot its meaning.
*Cliché*	这就是我常说的/这就是一个成语.
	This is just what I often say/This is just an idiom.
*“A just means A”*	我很高兴就是我很高兴.
	*wǒ-hěn-gāo-xìng* [I am very happy] just means *wǒ-hěn-gāo-xìng* [I am very happy].”
**Example**	
*Give an example*	他通过了考试, 真是’出人意料.’.
	He passed the exam; it is truly *chū-rén-yì-liào* [beyond all expectations].
**Interpretation**	
*Interpret the meaning*	‘一见钟情’就是第一次见面就爱上了彼此.
	*yī-jiàn-zhōng-qíng* [fall in love at first sight] means falling in love with each other during the first meeting.
*Interpret the context of usage*	‘不敢当’ 就是别人夸你的时候你说, 谦虚的.
	*bù-gan-dǎng* [I truly don’t deserve this] is what you say when people praise you in order to be modest.
**Association**	
*Associate a synonym/antonym*	‘等不及’就是’不耐烦.’.
	*děng-bù-jí* [can’t wait] is just *bu-nai-fan* [impatient]
*Associate another FS*	没听过’好意思,’ 只听过’不好意思.’.
	Haven’t heard of *hǎo-yì-si* [have the nerve], only heard of *bù-hǎo-yì-si* [be ashamed of].
*Associate L1*	对, 韩语里也有这样的说法.
	Correct. Koreans also have this saying.
**Metalinguistics**	
*Syntactic analysis*	对，是动词加补语的结构..
	Correct. It is a verb plus a complement structure.
*Semantic analysis*	应该是表达和自己的情绪有关系,因为有哭和笑两个字.
	It should be related to one’s emotions, because there is a *kū* [cry] and *xiào* [smile] in it.
*Pattern analysis*	‘谈天说地,’ 对的, 中文里说’天’就一定要说’地’.
	*tán-tiān-shuō-dì* [talk of everything under the sun], correct. In Chinese, when *tán* [sky] is mentioned, *dì* [earth] must also be mentioned.

Two trained research assistants coded approximately 20% of the think-aloud data (*n* = 750) independently. The intercoder reliability reached 95.3% (Cohen’s Kappa = 0.829). The agreement was considered high enough for one assistant to code the remaining data alone.

### Preliminary Data Processing

To analyze processing accuracy, we included all judgment data. Because the selected testing materials were highly familiar to NS participants and most non-native participants, the dichotomous judgment data (processing accuracy) were supposed to be positively skewed (Kurtosis-3 = 7.65, *p* < 0.000; with an expected kurtosis value of 3). To analyze processing speed, we transformed the raw response time data by log10 to reduce skewness (Kurtosis-3 = 0.11, *p* = 0.182). We excluded incorrect responses to target items, which led us to remove 1.9% of the NS data, 5.7% of the SL data, and 16.3% of the AL data. We further trimmed the extreme data by setting the cutoffs at three standard deviations from each participant’s mean response time. This procedure removed an additional 1.1% of the NS data, 0.7% of the SL data, and 0.6% of the AL data. To analyze processing strategies, we included all think-aloud data (Kurtosis-3 = 0.06, *p* = 0.417).

Processing accuracy was analyzed using a generalized linear mixed-effects model, and processing speed using a linear mixed-effects model in R via the lmerTest package. When we fitted the models, we included the variables of group, type of FSs, length of FSs, and their interactions as fixed effects. The participants and items were added as random effects beginning with a maximal structure encompassing random intercepts for participants and items, and random slopes for all fixed effects. The maximal random-effects structure was reduced when the model failed to converge. Regarding the above analyses, we report the model structure, the Wald chi-square statistics for processing accuracy, *F* statistics for processing speed, degrees of freedom, and the significance of the fixed effects. The full model outputs, including all results of pairwise contrasts, are available in the Supplementary Materials (Tables B and C). The processing strategy was cross-tabulated by group and type of FS to explore the frequency distribution. We also performed qualitative analysis of each strategy used by each group.

## Results

### RQ1: Processing Accuracy

RQ1: *Do ALs, SLs, and NSs demonstrate different patterns in FS processing accuracy*? To answer this question, we examined the yes-or-no judgments made by the three groups of participants on the speedy acceptability judgment task. First, we cross-tabulated the accuracy percentages by the three groups on different type and length conditions. The results (in Table 3) show that the judgment accuracy on all FS conditions increased as proficiency level increased. NSs’ judgments of idioms were either as accurate as, or more accurate than, their judgments of non-idioms. In contrast, SLs’ and ALs’ judgments of idioms were either as accurate as, or less accurate than, those of non-idioms.

**TABLE 3:** Mean judgment accuracy ratios by group and FS type and length.

		NS		SL		AL	
		Mean	SD	Mean	SD	Mean	SD
*Type*	*Length*						
Idiom	3-Character	0.98	0.13	0.93	0.26	0.79	0.41
	4-Character	1.00	0.00	0.94	0.25	0.84	0.37
Non-idiom	3-Character	0.98	0.14	0.97	0.18	0.88	0.33
	4-Character	0.96	0.18	0.94	0.24	0.85	0.36
Total		0.98	0.11	0.94	0.23	0.84	0.37

The dichotomous judgment data were then analyzed via a generalized linear mixed-effects model in R using the lmerTest package. When fitting the models, we entered the variables of group, length, and type as fixed effects. We added the participants and items as random effects, including the random intercepts of participants and items and the random slopes for all fixed effects. When dealing with the multicollinearity issue, we excluded the three-way interaction of group × type × length from the model because the two-way interactions and the planned *post hoc* contrasts for the two-way interactions were informative enough about the source of the effect of type and length on different group conditions. The analysis returned a significant effect for group (χ^2^ = 49.16, *df* = 2, *p* < 0.000) and the interaction of group × type (χ^2^ = 10.13, *df* = 2, *p* = 0.006) and type × length (χ^2^ = 9.54, *df* = 1, *p* = 0.002). The effect of type was marginally significant (χ^2^ = 2.91, *df* = 1, *p* = 0.088), while the effect of length (χ^2^ = 1.66, *df* = 1, *p* = 0.198) and the group × length interaction (χ^2^ = 1.42, *df* = 2, *p* = 0.493) were both non-significant.

We then conducted the planned pairwise comparisons. The analyses for the effect of group show that the judgment accuracy was significantly different between NSs and ALs (*p* < 0.000), NSs and SLs (*p* < 0.000), and ALs and SLs (*p* < 0.001). NSs’ judgments were more accurate than those of the two groups of learners, and SLs’ judgments were more accurate than those of the ALs. The analyses for the effect of type × group indicate that idioms were judged significantly more accurately than non-idioms by NSs (*p* = 0.012), while idioms were judged significantly less accurately than non-idioms by ALs (*p* = 0.024). SLs’ judgments of idioms and non-idioms were only marginally different (*p* = 0.061). This finding suggests that NSs’ recognition of idioms was more successful than their recognition of non-idioms, implying that idioms were more deeply entrenched in the NSs’ lexicons and therefore unlikely to be recognized incorrectly. For CSL learners with a relatively low proficiency level, the results hint that they were better at judging semantically transparent or syntactically regular sequences than the irregular formulae. For learners with a higher proficiency level, their judgment ability of the irregular formulae developed and approached their judgment ability of the regular formulae. The analyses for the effect of length × group demonstrated no significant results for NSs (*p* = 0.623), SLs (*p* = 0.297), or ALs (*p* = 0.747), thereby suggesting that longer FSs were recognized as successfully as shorter FSs by all three groups of participants.

### RQ2: Processing Speed

RQ2: *Do ALs, SLs, and NSs demonstrate different patterns in FS processing speed?* We used the eligible response time data to answer this question. We first examined the raw response times descriptively (results in Table 4). As expected, the mean response time under all FS conditions decreased as proficiency level rose. For the NS group, 3-C and 4-C idioms were both processed more quickly than their non-idiom counterparts. We noted a similar trend in the SL group. In contrast, for the AL group, 3-C and 4-C non-idioms were processed more quickly than their idiomatic counterparts. The fastest FS category was 4-C idioms for NSs (1000 ms), 3-C idioms for SLs (1516 ms), and 3-C non-idioms for ALs (2,345 ms).

**TABLE 4:** Raw RT (ms) by group, type of FSs, and length of FSs.

		NS			SL			AL		
		Mean	*N*	SD	Mean	*N*	SD	Mean	*N*	SD
*Type*	*Length*									
Idiom	3-Character	1199	303	825	1516	286	945	2415	243	1713
	4-Character	1000	311	369	1769	290	1360	2872	259	1781
Non-idiom	3-Character	1216	302	687	1652	299	1210	2345	272	1408
	4-Character	1205	296	833	1853	292	1674	2403	262	1379
*Average across types and lengths*		*1155*	*303*	*679*	*1698*	*292*	*1297*	*2509*	*259*	*1570*

The log10-transformed response time data were then sent into a linear mixed-effects model with group, type of FSs, and length of FSs included as fixed effects. We added the participants and items as random effects, beginning with a maximal random effects structure encompassing random intercepts for participants and items, and random slopes for all fixed effects. The outcomes showed a clear effect for group (*F* = 51.45, *df* = 2, *p* < 0.000), length (*F* = 8.90, *df* = 1, *p* = 0.003), the interaction for group × type (*F* = 7.62, *df* = 2, *p* < 0.001), group × length (*F* = 14.42, *df* = 2, *p* < 0.000), and group × type × length (*F* = 5.35, *df* = 2, *p* = 0.005). The effect of type (*F* = 0.96, *df* = 1, *p* = 0.327) and the interaction for type × length (*F* = 1.37, *df* = 1, *p* = 0.242) were non-significant.

To explore the differences between the levels of each variable, we performed planned pairwise comparisons. The analyses for the effect of group showed that processing speed was significantly different between NSs and ALs (*p* < 0.000), NSs and SLs (*p* = 0.017), and ALs and SLs (*p* < 0.000). Further, NSs outperformed the two learner groups, and the SLs outperformed the ALs. The analyses for the effect of group × type revealed that idioms and non-idioms were processed at a significantly different speed by NSs (*p* = 0.002), with idioms being processed faster than non-idioms, and by ALs (*p* = 0.019), with idioms being processed more slowly than non-idioms, but the difference was non-significant for SLs (*p* = 0.268). This pattern indicates that learners with lower proficiency might spend more cognitive effort processing semantically or syntactically irregular formulae than regular ones. As proficiency approached the near-native level, the cognitive efforts spent on irregular forms decreased, thereby generating comparable processing speed for idioms and non-idioms. For NSs, the processing pattern was reversed, as idioms were processed more effortlessly than non-idioms, suggesting that idioms might be pre-listed in NSs’ mental lexicons. The analyses for the effect of group × length showed that the processing speed of 3-C versus 4-C FSs was significantly different for the NS group (*p* = 0.002), with 4-C FSs being processed faster than 3-C FSs. A significant length effect was also found for the SL group (*p* = 0.002) and the AL group (*p* = 0.002). However, both with 3-C FSs were processed more quickly than 4-C FSs. The findings of the length effect for SLs and ALs were explainable, as the longer sequences required a longer time to read. Notwithstanding, the finding for NSs was counterintuitive. We computed the planned pairwise comparison for length × type for the NS group. The length effect existed only in the processing of idioms, with 4-C idioms responding significantly faster than 3-C idioms (*p* = 0.011), but the difference between 3-C non-idioms and 4-C non-idioms was non-significant (*p* = 1.00).

### RQ3: Processing Strategies

**FIGURE 1:**
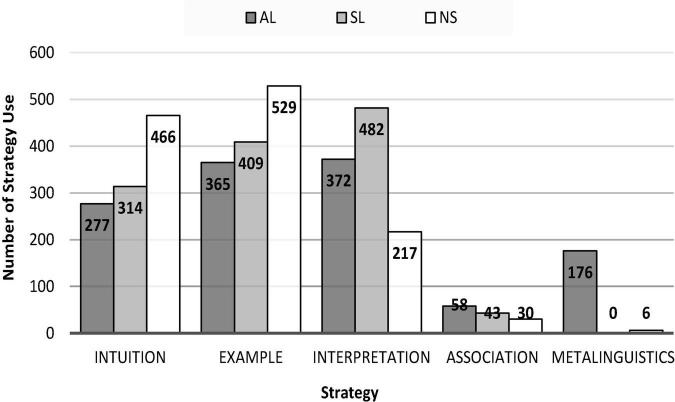
Frequencies of strategy use by group.

RQ3: *Do ALs, SLs, and NSs demonstrate different patterns in their FS processing strategies?* We first addressed this question by cross tabulating the frequency distributions of the five strategies (intuition, example, interpretation, association, and metalinguistics) by group. The results are presented in Figure 1. There is a clear group difference in strategy use. The most frequent strategy in the NS group was the example strategy, but the most frequent strategy in the AL and SL groups was the interpretation strategy. The intuition strategy was also more frequently used by NSs than it was by the two groups of learners. The association strategy was seldom used by all three groups. The metalinguistics strategy was almost exclusively used by ALs. To further explore whether there was a difference between each group’s strategy use for idiomatic versus non-idiomatic FSs, we cross-tabulated strategy use by type and group (see Table 5) and performed a qualitative analysis for each strategy.

**TABLE 5:** Crosstabulation of strategy by group and type.

	NS		SL		AL	
	Idiom	Non-idiom	Idiom	Non-idiom	Idiom	Non-idiom
	*n* (%)	*n* (%)	*n* (%)	*n* (%)	*n* (%)	*n* (%)
Intuition	242 (38.8)	224 (35.9)	139 (22.3)	175 (28.0)	156 (25.0)	121 (19.4)
Example	196 (31.4)	333 (53.4)	124 (19.9)	285 (45.7)	112 (17.9)	253 (40.5)
Interpretation	165 (26.4)	52 (8.3)	334 (53.5)	148 (23.7)	225 (36.1)	147 (23.6)
Association	20 (3.2)	10 (1.6)	27 (4.3)	16 (2.6)	40 (6.4)	18 (2.9)
Metalinguistics	1 (0.2)	5 (0.8)	0 (0.00)	0 (0.00)	91 (14.6)	85 (13.6)
Total	624 (1.00)	624 (1.00)	624 (1.00)	624 (1.00)	624 (1.00)	624 (1.00)

#### Intuition

Intuition was used more frequently by NSs; 38.8% of idioms and 35.9% of non-idioms were processed with intuition by NSs. SLs used intuition for 22.3% of idioms and 28% of non-idioms. ALs used intuition for 25% of idioms and 19.4% of non-idioms. When using intuition with idioms, NSs often immediately identified the idioms, saying, “*It is just an idiom.*” Learners either relied on their feelings or confessed that “*I have learned this but forgotten its meaning.*” When using intuition with non-idioms, all three groups of participants provided a cliché type of answer, such as “*A just means A*” or “*It is just what I often say*” without further elaboration. That NSs could quickly recognize idioms suggests that idioms are a very salient FS type in NSs’ mental lexicons. In contrast, learners’ feelings and confessions imply that idioms might not have been fully acquired. The cliché reports hint that both NSs and learners’ non-idiomatic FS knowledge had become proceduralized.

#### Example

The example strategy was more frequently used for non-idioms than idioms by all three groups. NSs provided examples for 53.4% of non-idioms, SLs for 45.7% of non-idioms, and ALs for 40.5% of non-idioms. For idioms, an example strategy was used 31.4% of the time by NSs, 19.9% of the time by SLs, and 17.9% of the time by ALs. In using the example strategy, the participants either described a typical situation in which an FS often occurs or directly used the target FS to make a sentence. Being able to use an FS in a real situation is evidence of the possession of proceduralized knowledge (DeKeyser, 2003). The prevailing use of the example strategy for non-idioms indicates that non-idiomatic FS knowledge has been fully acquired as communicative and productive knowledge. When the example strategy was used for idioms by learners (and occasionally by NSs), the examples often contained errors, denoting that their productive idiom knowledge was limited. Below are two incorrect examples for the same idiom.

*Target idiom*: 脱口而出 *cast-mouth-and-out* “*blurt out*”

An NS’s report: “我脱口而出吐了一口痰. (*I ‘tuo-kou-er-chu’ and spit sputum.)*”

An AL’s report: “嗯，这个，看起来是对的。比如说，嗯，我、我、我好久都没有跟你说什么，终于 脱口而出. (*Er, this one, seems correct. For example, I, I, I haven’t said anything to you for a long time, and finally ‘tuo-kou-er-chu’).*”

#### Interpretation

The interpretation strategy was the most frequent approach for both the AL and SL groups. SLs used interpretation for 53.5% of idioms and 23.7% of non-idioms, and ALs used interpretation for 36.1% of idioms and 23.6% of non-idioms. For NSs, 26.4% of idioms and 8.3% of non-idioms were interpreted. Interpretations include two types: one is to provide a correct interpretation for a familiar FS (which is the case for all NSs’ interpretations); the other is to propose an interpretation for an unfamiliar FS (which is the case for some learners’ interpretations).

In the latter case, learners are often based on the constituent word’s meaning and their logical thinking to make sense of the unknown FS, sometimes resulting in an erroneous interpretation. An example is shown below.

*Target idiom*: 千方百计 *thousand-method-hundred-plan* “*to make every possible effort to*”

An AL’s report: “是对的, 应该.…… 我觉得解释应该是有很多计划. (*Correct. Should*…*I think that the meaning should be ‘having many plans?’)*”

#### Association

This strategy was barely used by all three groups (all <7%). When employing this strategy, both NSs and the two groups of learners would associate synonyms or antonyms, but only learners confused the target FS with another Chinese FS with a similar form, but not exactly the same meaning (e.g., 一路顺风 one-road-smooth-wind “*wish you a happy voyage*” vs. 一帆风顺 one-sail-wind-smooth “*wish everything go smoothly*”). Learners also associated a similar expression in their L1. The association strategy suggests that learners store their FS knowledge in a network fashion (Fitzgerald et al., 2020). Thus, by activating one item, other related items are also activated. However, the learners’ think-aloud evidence suggests that such association may facilitate FS recognition but not necessarily FS comprehension or production.

#### Metalinguistics

The metalinguistics strategy was almost exclusively used by ALs in the processing of both idioms (14.6%) and non-idioms (13.6%). NSs employed metalinguistic strategies less than 1% of the time, and SLs used zero metalinguistic strategies. By scrutinizing the think-aloud reports, we found that NSs’ metalinguistic cases often involved questioning whether a lexical bundle (e.g., 不可能有 *not-may-possible-have* “*it is impossible to have*”) was legitimate to be used in isolation. Metalinguistic analysis is often considered evidence of accessing explicit knowledge (Ellis and Loewen, 2007). In this study, ALs’ metalinguistic analysis often occurred to unfamiliar FSs, suggesting that when FSs had not been acquired, learners would reply on their rule-based semantic and syntactic knowledge to make the judgments. The correct yes-or-no judgment indicates that the rule-based knowledge is useful to some extent, but the incorrect inference of the meaning suggests that the acquisition of FSs cannot depend on the learning of rules. The following examples display the incorrect metalinguistic analyses.

*Target idiom*: 谈天说地 *talk-sky-say-earth* “*talk of everything under the sun*”

An AL’s report: “应该是对的, 因为谈和说有关的, 天和地有关的, 但是怎么用……不知道. (*Should be correct, because ‘tan [talk]’ is related to ‘shuo [say]’; ‘tian [sky]’ is related to ‘di [earth],’ but how to use it*……*I don’t know.)*”

*Target non-idiom*: 打死了 *beat-die-ASPECT* “*beat to death*” An AL’s report: “对的.程度很深的时候用‘死了,’ 就是很厉害地打. (*Correct. ‘si-le [dead]’ is to indicate a deep degree,’ just meaning to beat severely.)*”

## Discussion

### Findings

This study examined the processing of Chinese idiomatic and non-idiomatic FSs among three groups of participants with different levels of proficiency. Due to the relatively small sample size, the findings should be considered to be tentative answers to our three research questions.

#### RQ 1

This question aimed to compare AL, SL, and NS processing patterns through dichotomous judgment data. The analysis of the dichotomous judgments returned significantly different processing patterns among the three groups. NSs judged idioms more successfully than they judged non-idioms. ALs judged non-idioms more successfully than they judged idioms. SLs judged non-idioms slightly more accurately than they judged idioms, but the difference was marginal. The length effect was absent in all three groups. These patterns suggest that idioms are more deeply entrenched in NSs’ long-term memory than non-idioms and are thus unlikely to be recognized as wrong. For ALs, both types of FSs might not have been fully acquired, but since non-idioms are semantically and syntactically transparent forms, their correctness is easier to judge than idioms. SLs were almost equally successful with idioms and non-idioms, indicating that in the super-advanced stage, learners’ idiomatic knowledge improved compared to that in the advanced stage.

#### RQ 2

This question aimed to compare AL, SL, and NS processing patterns through reaction time data. For NSs, idioms were processed significantly faster than non-idioms, regardless of the length of the FSs. In the AL group, we observed a reversed pattern; non-idioms were processed significantly faster than idioms were. Longer FSs took a significantly longer time to process than shorter ones did. For SLs, idioms were processed as fast as non-idioms, but longer FSs still required significantly longer processing times than shorter FSs. The presence of a length effect in the AL and SL groups suggests that learners were likely to read both idioms and non-idioms in a word-by-word fashion. For ALs whose idiom knowledge was quite limited, judging the correctness of an idiom may have largely relied on the online computation of the constituent words. Because idioms are semantically non-transparent forms, computing and making sense of an idiom would require more time than computing and making sense of a transparent non-idiom. However, SLs who possess better idiom knowledge still need to read an idiom word-by-word in the initial stages, but after they have reached the idiom key—the constituent word that determines when an idiom form can be recognized as an idiom (Cacciari and Tabossi, 1988)—they are able to judge the correctness without having to compute the rest of the sequence, hence saving processing time, resulting in a comparable processing speed for idioms and non-idioms. Overall, the SL performance exhibited a transition-stage pattern, gradually departing from the ALs and approaching NSs.

#### RQ 3

This question aimed to compare AL, SL, and NS processing patterns through think-aloud data. We coded think-aloud statements for five levels of processing strategies. The frequency analysis showed that the two learner groups used different processing strategies from the NS group. NSs preferred to use the example strategy, while learners preferred to use the interpretation strategy. This pattern suggests that the nature of NSs and learners’ formulaic knowledge may be fundamentally different. Being able to provide examples indicates that NSs’ formulaic knowledge has become proceduralized. Providing interpretations implies that learners’ formulaic knowledge might still be descriptive (Basturkmen et al., 2002; DeKeyser, 2007). In addition, all three groups employed more example strategies with non-idioms than idioms, denoting that both NSs’ and learners’ productive idiom knowledge is limited to different extents.

In sum, the following processing patterns emerged from the analyses of processing accuracy, speed, and strategy. First, the participants’ processing accuracy and global processing speed increased along with their proficiency. Second, CSL learners’ idiom processing ability was generally lower than that of non-idiom processing ability, but they demonstrated an improving trend as their proficiency level increased. Third, CSL learners’ use of processing strategies did not change much as proficiency rose and demonstrated a categorical difference from NSs. Fourth, all three groups exhibited poorer productive idiom knowledge than productive non-idiom knowledge.

### Implications

#### Moving Beyond the Lexical Plateau

In the analyses of processing accuracy and processing speed, we found that SLs’ processing patterns were significantly different from those of ALs. From the advanced to the super-advanced levels, the learners changed from being more successful at non-idiom processing to being equally successful at non-idiom and idiom processing, marking a transition toward NS performance (being more successful at idiom processing). This finding suggests that formulaic knowledge can grow substantially as learners move from an advanced to a higher proficiency level.

In the SLA literature, higher-than-advanced learners have long been overlooked. A major reason for this is that the number of achievers is very small, especially for learners of a non-English language such as Chinese. Another reason may have to do with the general assumption of the lexical plateau (see Daller et al., 2013 for an overview). Motivated by general learning curve models, L2 researchers have found that the development of lexical knowledge follows the general learning curve with slow growth at the beginning, followed by an accelerating phase and then flattening out to a plateau. This finding has led educators to assume that in the advanced stage, lexical knowledge probably ceases to grow because learners have already accumulated a large vocabulary inventory and sufficient L2 experience that allows them to successfully handle most target language scenarios. However, our findings imply that some lexical aspects maintain a fast-growing pace after learners have surpassed the advanced stage.

From a methodological angle, our findings hint that the lexical plateau phenomenon may be the outcome of the testing method used. That is, the instruments employed to test higher-than-advanced learners’ lexical knowledge might not be sufficient to reveal domain-specific progress. In recent years, as Chinese language education has received substantially more attention within and outside China (Gong et al., 2020a), the number of advanced learners has shown an incremental trend. In 2021, the newly published *Chinese Proficiency Grading Standards for International Chinese Language Education* (National Language Commission, 2021) changed the 6-level standard to a 9-level standard to accommodate Chinese learners’ increased overall proficiency (Ministry of Education of the People’s Republic of China, 2021). In contrast to this development, there is a profoundly inadequate understanding of higher-than-advanced Chinese learners and a severe dearth of teaching and testing materials. The findings about formulaic knowledge may inform researchers and teachers of where to direct their efforts to help advanced learners move beyond the lexical plateau.

#### Enhancing Idiomaticity in the Classroom

Another observation gained from the think-aloud data was that both NSs’ and learners’ ability to use idioms was limited to different extents. This was shown by the fact that all three groups used far fewer example strategies (ranging from 22% to 36%) with idioms than with non-idioms. Poor idiom competence was also reported by Lewis et al. (1998) and Yang and Xie (2013), who found that making mistakes in using idioms is a pronounced phenomenon in both native and non-native Chinese speakers.

As for why using idioms is problematic, there are multiple reasons for learning difficulties. The primary one is the semantic opaqueness of idioms. Because of semantic and syntactic idiosyncrasies, learning idioms largely depends on rote memorization (Long et al., 2018), which causes cognitive burdens for learners. Second, most idiomatic expressions are domain- or genre-specific (Xu and Qin, 2021), which means that language users may have passively encountered them multiple times, but have not had sufficient opportunities to actively use them (Ellis, 2012). As a result, there is a great gap between learners’ receptive and productive idiom knowledge. Laufer (1998) pointed out that the development of passive lexical knowledge does not imply the development of active lexical knowledge. Usage-based approaches also predict that true learning only occurs when a language form can be employed interactively (Abbot-Smith and Tomasello, 2006). The question then becomes: How can we increase the frequency of the interactive use of idioms? Since idioms are not frequently used in everyday communicative settings, we argue that L2 learners’ idiomaticity could be enhanced by integrating interactive activities into the classroom. Yang and Xie (2013) proposed a self-generated learning approach for Chinese idioms, the essential parts of which are that students work in pairs and make idiom-carrying sentences/stories, and then read and provide peer feedback on other groups’ productions. Through these ongoing input–output cycles, learners’ productive idiom knowledge becomes entrenched. The authors also stressed that teachers’ interventions, such as idioms solitaire, are needed throughout the process to maximize the learning-on-error effect.

#### Improving Formulaic Competence Outside the Classroom

Another finding we would like to consider is the processing strategy used by the three groups of participants. Although the processing accuracy and speed data demonstrate a trend whereby L2 learners’ FS knowledge increased as they moved from the advanced to the super-advanced levels, the learners’ processing strategy pattern basically remained unchanged. While NSs used the example strategy the most, both ALs and SLs employed the interpretation strategy the most. The use of examples indicates that NSs can interact with FSs in a communicative manner, which represents a high degree of knowledge mastery. In contrast, the interpretation strategy involves describing or inferring FSs’ meaning; both cases suggest that CSL learners’ formulaic knowledge is descriptive and static (Ellis, 2005; Bowles, 2011). The question we must ask then is: How can descriptive FS knowledge become communicative knowledge?

Dörnyei et al. (2004, p. 87) proposed that the acquisition of formulaic competence is a “socially loaded process” because formulaic expressions are so closely linked to the sociocultural reality of the target language. Thus, to incorporate descriptive formulaic knowledge into learners’ own language repertoires, learners must immerse themselves in host cultures through active participation in L2 communities. Taguchi et al. (2013) found that even when studying abroad, learners’ perceived encounters with formulae could not directly lead to gains in formulaic production, especially in the case of proficient learners. Moreover, learners’ contextually inappropriate use of formulae may occur due to a lack of socio-pragmatic knowledge. Gong et al. (2021) also suggested integrating opportunities and resources both in and outside the classroom to promote CSL/CFL learners’ Chinese proficiency. Based on previous literature and our observations, we assert that a more effective pedagogical means for cultivating formulaic competence—especially in the advanced stage—is to encourage students to step outside the classroom and to engage in everyday interactions with NSs. For teaching in CSL contexts, Gong et al. (2020b) recommended that language teachers adopt new pedagogical approaches that can provide sociocultural and psychological support to international students so that they become more willing to communicate with local Chinese people. For teaching in CFL contexts, Luo and Yang (2018, 2022) suggested that teachers create virtual exchange opportunities between foreign and Chinese students through telecollaboration, which is a mutually beneficial approach to promoting cultural learning and establishing the target language community.

## Limitations and Future Research

Although the experimental design enabled a controlled comparison of two types of formulae among three groups of Chinese speakers with different proficiency levels, a fact which had not been fully examined in the FS literature, we must acknowledge some limitations. First, the sample size of our study was relatively small; thus, the answers are more tentative than conclusive. Although higher-than-advanced learners are very scarce, endeavors could be made to conduct a larger-scale study.

Another issue is the homogeneity of the FSs. In this study, we could subdivide both idioms and non-idioms. For example, non-idiomatic FSs included lexical collocations and lexical bundles. According to Carrol and Conklin (2020), NSs’ processing of these two subtypes of FSs is regulated by different linguistic features. Thus, to more accurately depict the developmental path of L2 learners’ formulaic knowledge and to pinpoint the regulatory factors, finer-grained manipulation of FSs is needed.

Regarding the methodology of think-aloud protocols, there may be the issue of reactivity; i.e., the possibility that thinking aloud may alter participants’ cognitive processes (Bowles, 2010). Although substantial findings have confirmed that think-aloud protocols do not significantly influence speakers’ cognitive processes during relatively simple reading tasks (e.g., Bowles and Leow, 2005; Adrada-Rafael and Filgueras-Gómez, 2019), research using a counterbalanced think-aloud (versus silent) task order could eliminate potential suspects.

Another methodological issue concerns the use of NSs as the baseline group. According to our findings, NSs do not necessarily possess full idiom knowledge, especially productive idiom knowledge. Thus, future studies involving idiom production should carry out a preliminary test to confirm that the chosen NSs are eligible to serve as indexing norms.

## Data Availability Statement

The original contributions presented in the study are included in the article/Supplementary Material, further inquiries can be directed to the corresponding author.

## Ethics Statement

The studies involving human participants were reviewed and approved by University of Illinois at Urbana-Champaign. The patients/participants provided their written informed consent to participate in this study.

## Author Contributions

HZ designed the study, performed the experiments, analyzed the data, and wrote and edited the manuscript. BH analyzed the data, reviewed, and revised and edited the manuscript. JX supervised the research, analyzed the data, and reviewed the manuscript. All authors contributed to the article and approved the submitted version.

## Conflict of Interest

The authors declare that the research was conducted in the absence of any commercial or financial relationships that could be construed as a potential conflict of interest.

## Publisher’s Note

All claims expressed in this article are solely those of the authors and do not necessarily represent those of their affiliated organizations, or those of the publisher, the editors and the reviewers. Any product that may be evaluated in this article, or claim that may be made by its manufacturer, is not guaranteed or endorsed by the publisher.

## Footnotes

1
www.baidu.com
