# Supplementary material for: The Development of Formulaic Knowledge in Super-Advanced Chinese Language Learners: Evidence From Processing Accuracy, Speed, and Strategies
Source: Front Psychol. 2022 Mar 3;13:796784. doi: 10.3389/fpsyg.2022.796784 (PMC8928222; doi:10.3389/fpsyg.2022.796784)
Supplement: Supplementary file 1 [file Table_1.pdf]

Supplementary Material

Table A. Test material

	Idiom	Length	Lg10 frequency	Stroke	Rating	Non-idiom	Length	Lg10 frequency	Stroke	Rating
1	不敢当	3-C	7.03	21	4.18	不敢动	3-C	7.74	21	N/A
2	走后门	3-C	7.18	16	4.15	走出门	3-C	6.9	15	N/A
3	看热闹	3-C	7.5	27	4.21	看一遍	3-C	7.32	22	N/A
4	难为情	3-C	7.29	25	4.06	难度大	3-C	7.23	22	N/A
5	暗地里	3-C	7.48	26	4.03	被子里	3-C	6.98	20	N/A
6	来得及	3-C	7.61	21	4.79	来源于	3-C	7.69	23	N/A
7	看不起	3-C	7.67	23	4.79	看着他	3-C	7.68	25	N/A
8	开夜车	3-C	6.49	16	4.12	别的车	3-C	7.78	19	N/A
9	吹牛皮	3-C	6.9	16	4.33	吹走了	3-C	7.51	16	N/A
10	打官司	3-C	7.51	18	4.03	打死了	3-C	7.68	13	N/A
11	打交道	3-C	7.61	23	4.12	打麻将	3-C	7.51	25	N/A
12	出人命	3-C	7.69	15	4.09	出不去	3-C	7.91	14	N/A
13	看上去	3-C	7.6	17	4.73	看不见	3-C	7.8	17	N/A
14	过日子	3-C	7.55	13	4.45	过几天	3-C	8	12	N/A
15	不得已	3-C	7.64	18	4.06	不能做	3-C	8	25	N/A
16	不见得	3-C	8	19	4	看见你	3-C	7.38	20	N/A
17	等不及	3-C	7.48	19	4.61	等着他	3-C	8	28	N/A
18	好容易	3-C	7.67	24	4.15	好像要	3-C	7.72	28	N/A
19	半辈子	3-C	7.47	20	4.21	半年后	3-C	7.49	17	N/A
20	靠得住	3-C	7.47	33	4.18	靠窗的	3-C	6.95	35	N/A
21	好意思	3-C	7.49	28	4	好想吃	3-C	6.47	25	N/A
22	打主意	3-C	6.93	23	4.09	打桌球	3-C	6.51	16	N/A
23	慢半拍	3-C	6.99	27	4	慢下来	3-C	7.3	24	N/A
24	过得去	3-C	7.48	22	4.03	过多久	3-C	7.72	15	N/A
25	供不应求	4-C	7.52	26	4.21	供应产品	4-C	7.5	30	N/A
26	大吃一惊	4-C	7.51	21	4.88	大吃一顿	4-C	7.23	20	N/A
27	轻而易举	4-C	7.52	32	4.36	轻松自在	4-C	6.98	30	N/A
28	无可奈何	4-C	7.58	24	4.48	有可能是	4-C	8	30	N/A
29	一无所有	4-C	7.67	19	4.7	一定会有	4-C	8	21	N/A
30	不择手段	4-C	7.49	25	4.06	重要手段	4-C	7.73	31	N/A
31	得不偿失	4-C	7.54	31	4	得到一个	4-C	7.68	23	N/A
32	不可或缺	4-C	7.64	27	4.24	不可能有	4-C	7.42	25	N/A
33	一见钟情	4-C	7.59	25	4.64	一段感情	4-C	7.59	34	N/A
34	哭笑不得	4-C	7.49	35	4.58	哭的时候	4-C	7.74	35	N/A
35	谈天说地	4-C	7.45	29	4.21	谈论一下	4-C	7.39	20	N/A
36	出人意料	4-C	7.5	30	4.67	出门在外	4-C	7.58	19	N/A
37	不约而同	4-C	7.52	22	4.36	不同的是	4-C	7.91	27	N/A

38	兴高采烈	4-C	6.81	34	4.7	我很高兴	4-C	7.1	32	N/A
39	脱口而出	4-C	7.49	25	4.18	忽然长出	4-C	7.11	24	N/A
40	心中有数	4-C	7.54	30	4.03	心里有事	4-C	7.65	35	N/A
41	千方百计	4-C	7.55	17	4.18	千万不要	4-C	7.51	19	N/A
42	一帆风顺	4-C	7.57	20	4.7	一片风雨	4-C	6.97	17	N/A
43	一目了然	4-C	7.6	20	4.06	了解一下	4-C	7.74	19	N/A
44	天涯海角	4-C	7.5	32	4.06	天气真好	4-C	6.66	24	N/A
45	心甘情愿	4-C	7.53	34	4.3	心爱的人	4-C	7.96	24	N/A
46	迫不及待	4-C	7.61	24	4.36	看不下去	4-C	7.6	35	N/A
47	无能为力	4-C	7.55	20	4.45	谁能告诉	4-C	7.79	27	N/A
48	前所未有	4-C	7.65	28	4.15	不是所有	4-C	7.41	26	N/A

Table B. Full Models' Outputs of RQ1 (Processing Accuracy)

Results of the generalized linear mixed effects model

	Estimate	***SE***	***z***	***p***
Fixed effects				
(Intercept)	4.738032	0.509705	9.29564	1.46E-20
groupG2	-3.4449	0.540226	-6.37679	1.81E-10
groupG3	-2.0436	0.557852	-3.66334	0.000249
typeT2	-0.89125	0.522683	-1.70514	0.088169
lengthL2	0.614941	0.477861	1.286862	0.198142
typeT2:lengthL2	-0.79738	0.258211	-3.08808	0.002015
groupG2:typeT2	1.644092	0.536739	3.063115	0.00219
groupG3:typeT2	1.760978	0.572498	3.075957	0.002098
groupG2:lengthL2	-0.16594	0.468405	-0.35427	0.723136
groupG3:lengthL2	-0.47603	0.508928	-0.93537	0.3496
Random effects	Variance	SD		
subj				
(Intercept)	0.289	0.5376		
item				
NA				

Results of Type III Wald chi-square tests

	Chi-square	***df***	***Sig.***
(Intercept)	86.4089	1	< 2.2e-16
group	49.1661 11 ***	2	2.107e-
type	2.9075	1	0.088169
length	1.6560	1	0.198142
type:length	9.5362	1	0.002015
group:type	10.1348	2	0.006299
group:length	1.4153	2	0.492789

Pairwise contrast				
Contrast	***Estimate***	***SE***	*z*	*p*
Group				
G1 - G2	2.71	0.355	7.624	<.0001
G1 - G3	1.40	0.363	3.864	0.0003
G2 - G3	-1.30	0.266	-4.904	<.0001
Type*Group				
G1: T1 - T2	1.290	0.513	2.513	0.0120
G2: T1 - T2	-0.354	0.157	-2.263	0.0237
G3: T1 - T2	-0.471	0.251	-1.874	0.0610
Length*Group				
G1: L1 - L2	-0.2163	0.439	-0.492	0.6225
G2: L1 - L2	-0.0503	0.156	-0.322	0.7474
G3: L1 - L2	0.2598	0.249	1.044	0.2967

Table C. Full Models' Outputs of RQ2 (Processing Speed)

**Results of the general linear mixed effects model**				
	Estimate	***SE***	*t*	*p*
Fixed effects				
(Intercept)	3.024015	0.03456	87.50014	5.50E-51
Group: AL	0.298112	0.044527	6.695126	1.79E-08
Group: SL	0.089106	0.04426	2.013242	0.049611
Type: nonidiom	0.015476	0.01509	1.025543	0.305181
Length: 4-C	-0.04658	0.014978	-3.10983	0.001888
Group: AL x Type:4-C	-0.0096	0.022307	-0.43055	0.666825
Group: SL x Type: 4-C	0.004299	0.021528	0.199684	0.84174
Group: AL x Length: 4-C	0.129181	0.022382	5.771698	8.56E-09
Group: SL x Length: 4-C	0.089991	0.021534	4.179007	3.00E-05
Type: nonidiom x Length: 4-C	0.036759	0.021324	1.723791	0.084838
Group: AL x Type: nonidiom x Length: 4-C	-0.10278	0.031464	-3.26653	0.0011
Group: SL x Type: nonidiom x Length: 4-C	-0.05223	0.030452	-1.71516	0.086408
Random effects	Variance	SD		
Intercept | Participants	0.013568	0.11648		
Intercept | Item	0.000889	0.02982		
Residual	0.034419	0.18552		

**Results of analysis of variance with satterthwaite's method**			
	F	***df***	***Sig.***
group	51.4539	2	4.192e-14
type	0.9611	1	0.32699-
length	8.8997	1	0.002873
group:type	7.6182	2	0.000500
group:length	14.4212	2	5.805e-07
type:length	1.3675	1	0.242321

group:type:length	5.3524	2	0.004777	
Pairwise contrast				
Contrast	***Estimate***	***SE***	***t***	***p***
Group				
G1 - G2	-0.332	0.0423	-7.861	<.0001
G1 - G3	-0.123	0.0422	-2.918	0.0172
G2 - G3	0.209	0.0248	8.443	<.0001
Type*Group				
G1: T1 - T2	-0.0339	0.0107	-3.174	0.0015
G2: T1 - T2	0.0271	0.0116	2.345	0.0191
G3: T1 - T2	-0.0120	0.0109	-1.108	0.2681
Length*Group				
G1: L1 - L2	0.0282	0.0107	2.644	0.0082
G2: L1 - L2	-0.0496	0.0116	-4.285	<.0001
G3: L1 - L2	-0.0357	0.0109	-3.283	0.0010
